# Development and validation of a nomogram to predict perioperative blood transfusion in patients undergoing total knee arthroplasty

**DOI:** 10.1186/s12891-020-03328-9

**Published:** 2020-05-20

**Authors:** Chuan Hu, Yuan-he Wang, Rui Shen, Chuan Liu, Kang Sun, Lin Ye, Jian-jun Ye, Xu Yang, Shao-qi Tian, Teng-bo Yu

**Affiliations:** 1 Department of Orthopaedic Surgery, The Affiliated Hospital of Qingdao University, Qingdao, 266071 China; 2 Department of Medical Oncology, The First Hospital of China Medical University, Shenyang, 110001 China; 3 Wenzhou Medical University, Wenzhou, 325000 China

**Keywords:** Transfusion, Risk factors, Total knee arthroplasty, Preoperative nomogram, Validation, Subgroup analysis

## Abstract

**Background:**

The need for a transfusion is one of the adverse events following total knee arthroplasty (TKA), and accurately predicting this need remains challenging for arthroplasty surgeons. The purpose of the present research is to study the preoperative predictors of transfusion risk in patients following TKA and develop a nomogram.

**Methods:**

The nomogram was developed based on a training set of 5402 patients who underwent TKA at the Affiliated Hospital of Qingdao University between September 2013 and November 2018. The independent predictors of transfusion were identified by univariate, LASSO, and binary logistic regression analyses. Then, a nomogram was established based on these independent predictors. The area under the curve (AUC), calibration curve, and decision curve analysis (DCA) were selected to evaluate the nomogram. The results were validated using an independent set of 1116 patients who underwent TKA between December 2018 and September 2019. In addition, we also carried out subgroup analyses in the training and testing sets based on the independent predictors.

**Results:**

Five independent predictors were identified by multivariate analysis and were used to establish the nomogram. The AUCs of the nomogram were 0.884 (95% CI: 0.865–0.903) and 0.839 (95% CI, 0.773–0.905) in the training and testing sets, respectively. In both the training and testing sets, the calibration curve indicated that the prediction by the nomogram was highly consistent with the actual observation, and the DCA indicated that the nomogram had a favorable level of clinical usefulness. In addition, the AUC of the nomogram was significantly higher than the AUC of any independent predictor for predicting transfusion risk following TKA, and the subgroup analysis showed good performance in 20 subgroups.

**Conclusion:**

Lower preoperative Hb levels, simultaneous bilateral TKA, lower BMI, older age, and coronary heart disease were identified as independent predictors of postoperative transfusion in patients following TKA. A nomogram incorporating the above five predictors could accurately predict the transfusion risk.

## Background

Total knee arthroplasty (TKA) is a cost-effective procedure for end-stage knee disease, with outcomes that have been confirmed by previous studies. However, due to perioperative blood loss, some patients require allogenic blood transfusion after TKA. The reported incidence of transfusion varies from 3.5–29.1% in TKA patients [1–3]. Although blood transfusion can increase the hemoglobin level and improve postoperative recovery, it is an important risk factor for many catastrophic complications, such as deep venous thrombosis [4], surgical site infection [5], and periprosthetic joint infection [6]. In addition, blood transfusion can lead to an increase in the length of stay and costs, which creates a larger economic burden for society [7].

Several strategies have been used to reduce the risk for transfusion in TKA patients, such as the use of a tourniquet and tranexamic acid [8, 9]. Unfortunately, the side effects of these strategies continue to worry orthopedists, and some patients still need blood transfusions. Therefore, it is necessary for us to recognize high-risk patients early for specific interventions that can reduce the transfusion risk following TKA.

Currently, nomograms are widely used as prognostic devices in medicine. In a nomogram, multiple risk factors can be combined to predict the probability of outcomes and visualize the results. However, no previous study has reported the predictive value of a nomogram for postoperative transfusion in patients following TKA. Therefore, our study aimed to investigate the incidence and preoperative risk factors for transfusion in patients following TKA and develop a preoperative nomogram for predicting transfusion risk following TKA.

## Methods

We retrospectively reviewed 6062 consecutive patients who underwent TKA from September 2013 to November 2018 at the Affiliated Hospital of Qingdao University (Shandong, China). The inclusion criteria were as follows: primary bilateral or unilateral TKA; no history of knee joint infection; and no history of transfusion within 1 month before TKA. The exclusion criteria were as follows: periarticular tumor; revision TKA; TKA combined with other operations simultaneously; staged bilateral TKA interval within 1 month; and incomplete data. Moreover, from December 2018 to September 2019, we prospectively included patients who underwent TKA in our hospital with the same inclusion and exclusion criteria to create the testing set.

The variables collected in our research are shown in Table 1. All data were independently collected from our hospital’s medical recording system by two arthroplasty surgeons, and any controversial data were agreed upon by the two doctors who extracted the data and a third independent arthroplasty surgeon. In addition, in our research, the transfusion group was defined as patients who received allogenic blood transfusion within 14 days after TKA. Moreover, it should be pointed out that the indication for transfusion in our institution is patients with a Hb < 70 g/L or patients with Hb < 80 g/L but with symptoms of anemia.

**Table 1:** Comparison of preoperative variables between the two groups

	Transfusion(*n* = 391)	Non-transfusion(*n* = 5011)	t/z/χ^2^	P
Age, years	67 (61,72)	66 (61,71)	1.656	0.098
Sex			17.307	0.000
Male	57	1192		
Female	334	3819		
BMI, Kg/m^2^	26.04 (23.52,29.14)	27.34 (24.88,29.69)	5.292	0.000
Duration of disease, years			11.731	0.008
<1	19	251		
1–3	29	562		
3–10	125	1816		
>10	218	2382		
Smoking	28	503	3.387	0.066
Alcohol	24	553	9.119	0.003
Allergies	65	754	0.701	0.402
Transfusion history	58	294	47.872	0.000
Blood type (Rh)			0.000	1.000
Rh+	389	4990		
Rh-	2	21		
Blood type (ABO)			3.748	0.290
A	101	1493		
B	121	1540		
O	124	1401		
AB	45	577		
CCI	3 (2,3)	3 (2,3)	1.065	0.287
Indications				0.155*
Osteoarthritis	372	4835		
Rheumatoid arthritis	16	151		
Traumatic arthritis	3	10		
Psoriatic arthritis	0	6		
Others	0	9		
Procedures			653.561	0.000
Bilateral TKA	231	570		
Unilateral TKA	160	4441		
TXA use	130	2060	9.345	0.002
Comorbidities				
Hypertension	186	2549	1.578	0.209
Diabetes mellitus	68	801	0.532	0.466
Coronary heart disease	67	687	3.544	0.060
Arrhythmia	9	139	0.303	0.582
Heart failure	1	6		0.471*
Other cardiac diseases	8	78	0.555	0.456
Peripheral vascular disease	8	114	0.086	0.769
Cerebrovascular disease	38	342	4.644	0.031
Mental illness	3	45	0.070	0,791
Pulmonary disease	9	180	1.789	0.181
Digestive system disease	22	359	1.308	0.253
Urinary system diseases	5	121	2.054	0.152
Gynecologic diseases	12	149	0.011	0.915
Laboratory tests				
RET, 10^9^/μl	61 (57,78)	64 (52,78)	3.517	0.000
MCV, fL	89.5 (86.4,92.6)	89.4 (86.7,92.2)	0.257	0.797
MCH, Pg	29.7 (28.6,30.7)	29.9 (28.9,30.9)	2.291	0.022
MCHC, g/L	332.0 (324.0,340.0)	333.0 (327.0,341.0)	3.563	0.000
Hb, g/L	80.0 (74.0,91.0)	105.0 (95.00,115.0)	23.463	0.000
RDW, %	13.0 (12.5,13.7)	12.8 (12.4,13.3)	23.046	0.000
PLT, 10^9^/L	146.0 (121.0,179.0)	179.0 (149.0,213.0)	11.825	0.000
PDW, fL	11.7 (10.8,13.2)	11.8 (10.8,13.0)	0.452	0.651

*TKA* Total knee arthroplasty, *BMI* Body mass index, *CCI* Charlson comorbidity index, *TXA* Tranexamic acid, *RET* Reticulocyte, *MCV* Mean corpuscular volume, *MCH* Mean corpuscular hemoglobin, *MCHC* Mean corpuscular hemoglobin concentration, *Hb* Hemoglobin, *RDW* Red blood cell distribution width, *PLT* Platelet, *PDW* Platelet distribution width

*Fisher's exact test

All patients included in our study underwent TKA that was performed by senior arthroplasty surgeons in our hospital following an identical protocol. A midline incision in the knee joint and medial parapatellar approach were used. Posterior Cruciate-Stabilizing prosthesis (NexGen LPS-Flex, Zimmer, Warsaw, IN) or Medial Pivot prosthesis (Advance Medial - Pivot Knee System, Wright Medical Group, Arlington, TN) were selected and fixed with cement. One intra-articular drainage line was used in each joint and removed within 48 h after TKA. When the joint capsule was closed, the patients who received TXA received 1 g TXA through the drainage line. All patients underwent rehabilitation exercises following a unified rehabilitation program, and rehabilitation work was guided by a professional therapist.

### Statistical analysis

All statistical analyses were performed with SPSS (version 24, IBM, USA) and R software (version 3.6.1, R Foundation for Statistical Computing, Austria). The normality of continuous variables was determined by the Kolmogorov-Smirnov test or the Shapiro-Wilk test in SPSS. The continuous data are presented as the mean ± SD or median (interquartile range), while the categorical data are presented as a number or percentage. In univariate analysis, Student’s t-test or Mann-Whitney U test was used for continuous variables, and the chi-square test or Fisher’s exact test was used for categorical variables. Predictors with a *P* value< 0.1 were included in the LASSO regression to avoid overfitting. Then, logistic regression analysis was performed to determine the independent predictors for transfusion in patients following TKA.

A nomogram was established based on the independent predictors in R software. The area under the curve (AUC) based on the receiver operating characteristic (ROC) curve was used to evaluate the discrimination of the nomogram. Furthermore, the calibration curve was used to evaluate the calibration of the nomogram, and decision curve analysis (DCA) was used to estimate the clinical usefulness of the nomogram in the training and testing sets. In addition, the ROC curves of each independent predictor were established, and comparisons of the AUCs between the nomogram and independent risk factors was performed. Finally, to confirm the predictive ability of the nomogram in subgroups, the patients were divided into subgroups according to the independent predictors, and the median of value of each continuous variable was used as the cutoff. The ROC curves of the nomogram and each independent predictor in each subgroup were generated, and the AUCs were identified. A *P* value< 0.05 (two-sided) was considered significant.

## Results

**Fig. 1:**
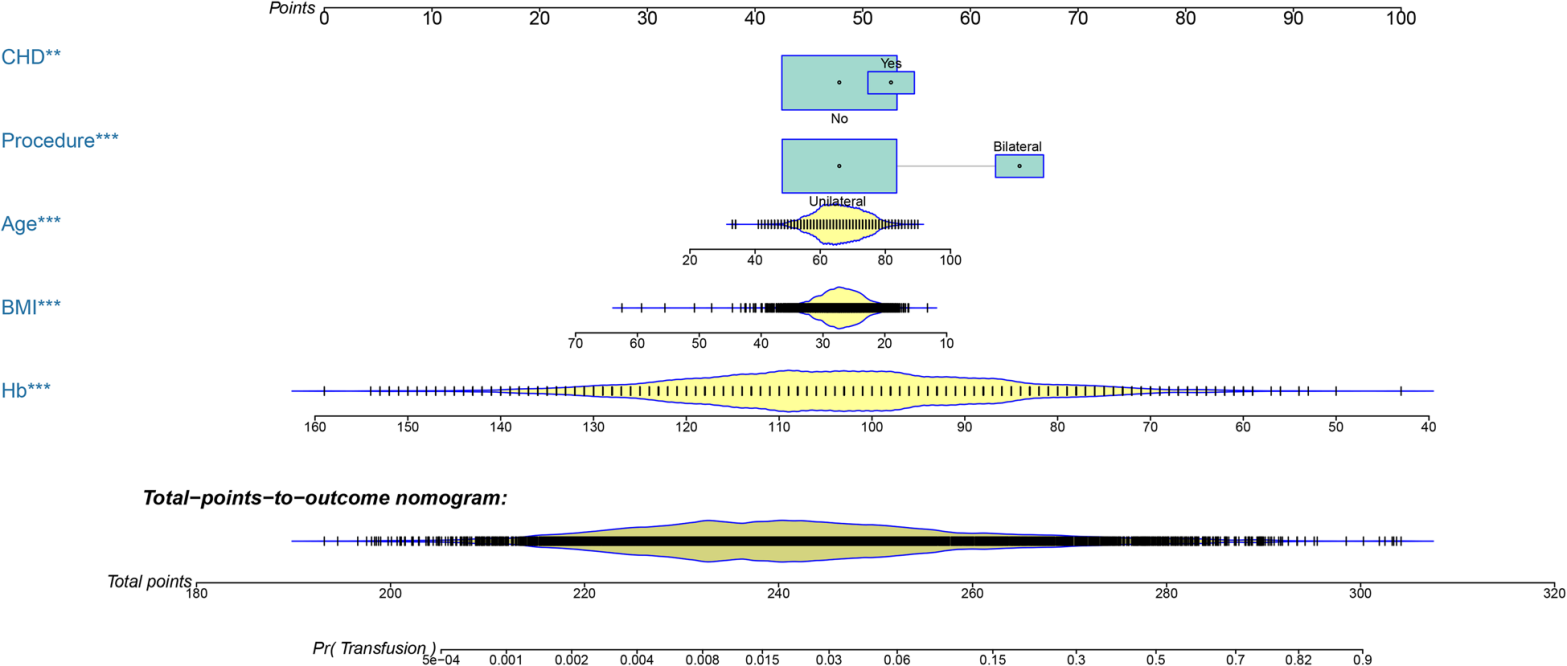
A nomogram based on independent risk factors for predicting transfusion risk following TKA. TKA: Total knee arthroplasty; CHD: Coronary heart disease; Hb: Hemoglobin; BMI: Body mass index

From September 2013 to November 2018, 6062 consecutive patients underwent TKA in the Affiliated Hospital of Qingdao University, and 5402 patients were included in the training cohort (Fig. 1). There were 1249 males and 4153 females. The mean age of the patients was 65.92 ± 7.38 years old (range: 33–90 years old), and the average body mass index (BMI) was 27.36 ± 3.92 kg/m^2^ (range: 13.11–62.50 kg/m^2^). Among these patients, 801 underwent simultaneous bilateral TKA, while 4601 underwent unilateral TKA.

### Independent risk factors for transfusion in the training set

In the training set, 391 patients received transfusions within 14 days after TKA, and the incidence of postoperative transfusion was 7.2%. Seventeen variables with a *P* value < 0.1 in the univariate analysis were included in the LASSO regression analysis, and fourteen variables were determined as significant predictors (Supplementary Fig. 1). Then, binary logistic regression analysis demonstrated that simultaneous bilateral TKA, coronary heart disease, Hb level, age, and BMI were independent predictors of postoperative transfusion following TKA (Table 2). The preoperative Hb value reported had the highest score, which suggested that this parameter had the greatest impact on the model.

**Table 2:** Multivariate logistic analysis of transfusion in patients following TKA

	B	SE	Wald	OR	95%CI	P
Age	0.031	0.009	12.288	1.032	1.014–1.050	0.000
BMI	−0.059	0.017	12.484	0.942	0.912–0.974	0.000
Procedure	1.725	0.133	167.779	5.613	4.324–7.287	0.000
CHD	0.495	0.169	8.605	1.641	1.179–2.285	0.003
Hb	−0.089	0.005	314.136	0.915	0.906–0.924	0.000

*TKA* Total knee arthroplasty, *BMI* Body mass index, *CHD* Coronary heart disease, *Hb* Hemoglobin

### Development and validation of a nomogram for predicting transfusion risk

**Fig. 2:**
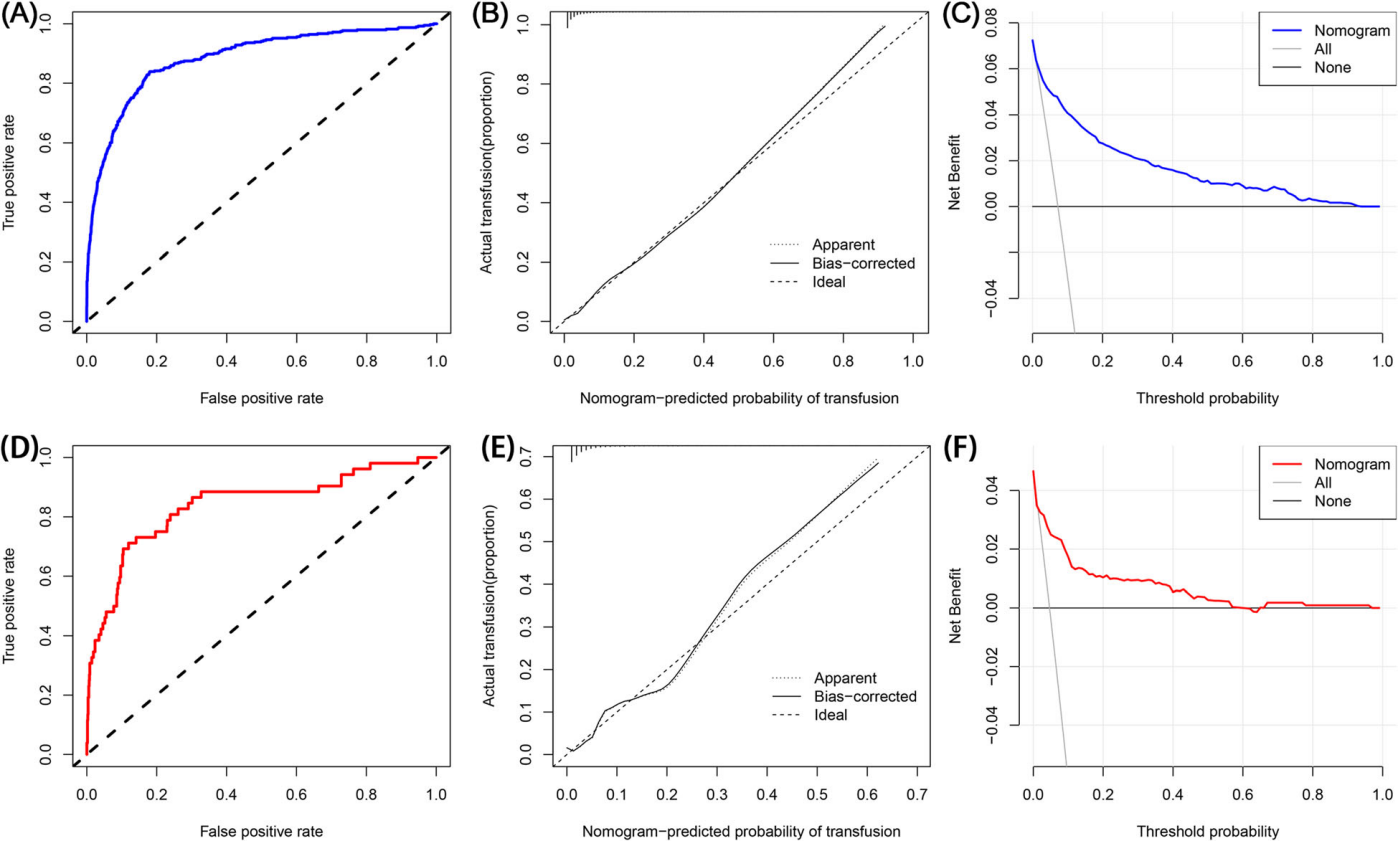
The receiver operating characteristic curve (**a**), calibration curve (**b**), and decision curve analysis (**c**) of training set. The receiver operating characteristic curve (**d**), calibration curve (**e**), and decision curve analysis (**f**) of testing set

Five independent predictors were used to establish a nomogram (Fig. 1), and the points of each variable are shown in supplementary Table 1. The AUC of our nomogram was 0.884 (95% CI, 0.865–0.903) in the training set (Fig. 2a), showing good accuracy in predicting the risk of transfusion in patients following TKA. The favorable calibration curve indicated that the prediction by the nomogram was highly consistent with the actual observation (Fig. 2b). Moreover, DCA showed that if the threshold probability of a patient and a doctor was higher than > 2 and < 94%, using this nomogram to predict transfusion risk adds more benefit than the scheme (Fig. 2c).

A total of 1116 patients were included in the testing set, and 52 patients received transfusions within 14 days after TKA. In the testing set, the AUC of the nomogram was 0.839 (95% CI: 0.773–0.905) (Fig. 2d), and the calibration curve showed good agreement between the prediction and observation regarding the probability of transfusion (Fig. 2e). Furthermore, the DCA demonstrated that if the threshold probability of a patient and a doctor was > 1 and < 62%, using the nomogram to predict postoperative transfusion has a net benefit (Fig. 2f).

### Comparison of the AUCs of the nomogram and a single factor for predicting transfusion risk

**Fig. 3:**
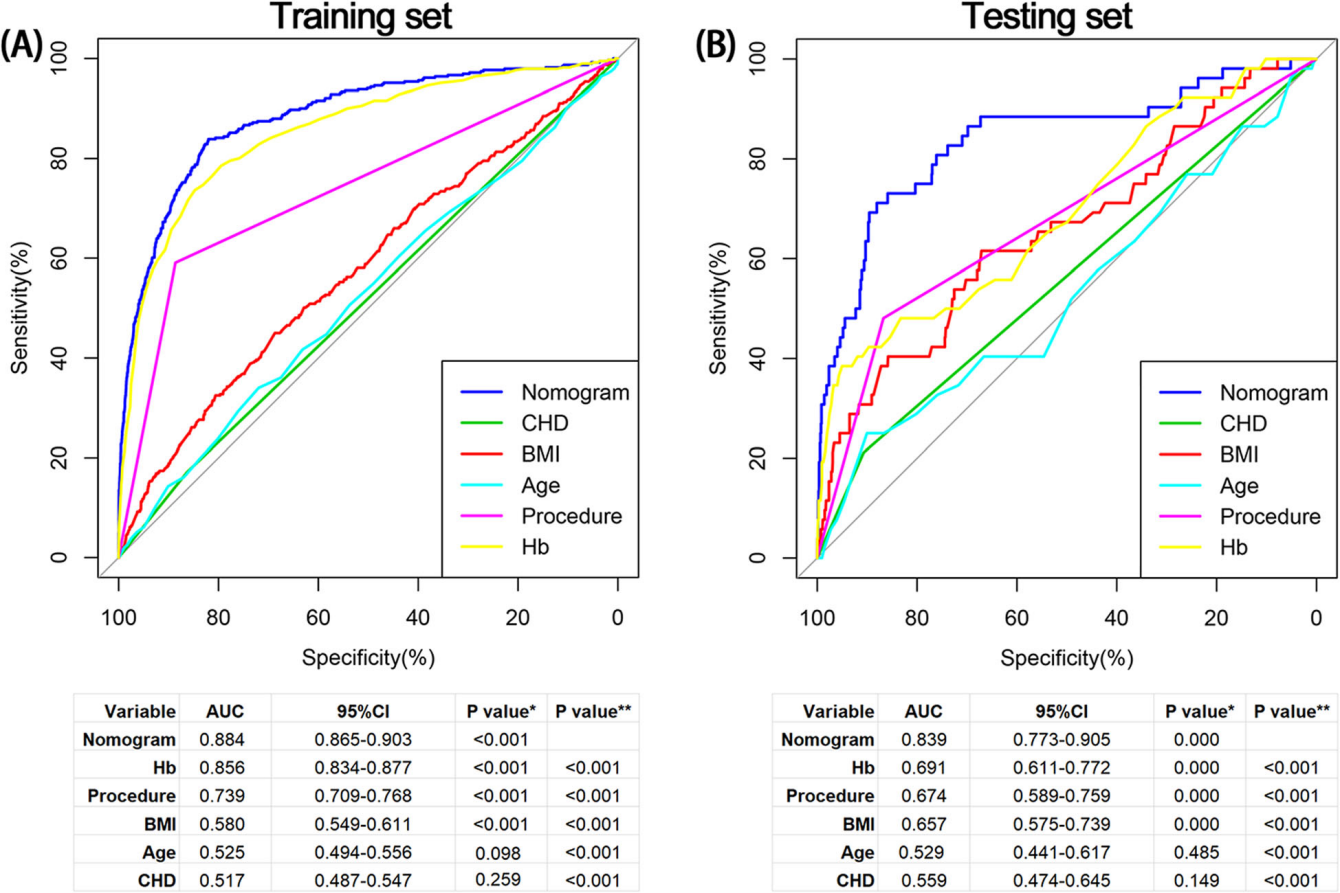
The receiver operating characteristic curves with corresponding area under the curves of nomogram and independent predictors in training set (**a**) and testing set (**b**). CHD: Coronary heart disease; Hb: Hemoglobin; BMI: Body mass index

The ROC curves of the nomogram, Hb, age, BMI, TKA procedure and CHD in the training set were generated (Fig. 3a). The results demonstrated that the AUC of the nomogram was significantly higher than the AUCs of each single independent predictor of transfusion risk in patients following TKA (all *P* values< 0.001) (Fig. 3a). Similar to the training set, in the testing set, the AUC of the nomogram was also significantly higher than the AUCs of each independent predictor in the testing set (all P values< 0.001) (Fig. 3b).

### Subgroup analysis of the nomogram

To determine the predictive performance of the nomogram in subgroups, the patients were divided into subgroups based on categorical variables or the median of independent predictors. We found that the nomogram can serve as a promising predictive tool for transfusion risk in patients following TKA in different subgroups, with AUCs ranging from 0.691–0.898 and 0.745–0.938 in the training set (Supplementary Fig. 2A- 2J) and testing set (Supplementary Fig. 2 K-2 T), respectively. More importantly, in most of the subgroups (8/10 in the training set and 5/10 in the testing set), the AUCs of the nomogram were significantly higher than those of each independent predictor.

## Discussion

In our study, a nomogram based on five preoperative predictors was established and confirmed as a tool that can accurately predict the postoperative transfusion risk of TKA patients. By using this nomogram, improved individualized patient counseling and decision-making regarding perioperative blood management for TKA patients can be achieved.

Although some predictive models have been established in previous studies, we think our study improves upon the previous work. In the predictive tools established by Noticewala et al. and Ahmed et al. [10, 11], only patients who underwent unilateral TKA were included. Conversely, in our study, patients who underwent unilateral and those who underwent bilateral TKA were included. More importantly, by subgroup analysis, we confirmed that our nomogram can perform well in both the unilateral TKA cohort and the bilateral TKA cohort. In addition, the advantages of our study compared with the predictive model generated by Jo et al. [12] are as follows. First, there was an improvement in the definition of the transfusion group. In the previous study, the definition of patients in the transfusion group was defined as patients with Hb < 70 g/L following TKA. Differently, in our study, patients who needed transfusions were defined as patients who actually received allogenic blood transfusion within 14 days after TKA. Although a Hb level of 70 g/L is the transfusion threshold that is adopted by many hospitals, some patients do not need to receive blood transfusions when their Hb < 70 g/L [13]. In addition, a recent study indicated that transfusion thresholds can be Hb values greater than 70 g/L during actual clinical practice [14]. Therefore, defining transfusion as patients with Hb < 70 g/L may cause a discrepancy with the criteria in clinical practice.

In our research, five independent variables were confirmed as predictors of transfusion. Their relationship to transfusion has been extensively reported in previous studies. Preoperative Hb level is one of the most important predictors of postoperative transfusion in patients following TKA, for both unilateral or simultaneous bilateral TKA [1, 2]. In addition, a recent study showed that preoperative Hb levels can still be used as an important predictor for postoperative transfusion in patients with TKA using antifibrinolytic drugs [15]. Interestingly, the use of tranexamic acid was not determined to be an independent predictor of blood transfusion in our study. However, a recent study reported that the use of tranexamic acid can effectively reduce the transfusion risk in patients undergoing unilateral TKA [9]. Unfortunately, the management of tranexamic acid can result in different outcomes, and there is no consensus on how to use tranexamic acid for TKA [16, 17]. Therefore, the dosage, administration route and administration time need to be further studied in future research.

Compared with those undergoing unilateral TKA, patients undergoing simultaneous bilateral TKA have a higher risk of transfusion. Similar results have been described in previous studies, and longer operation times and more perioperative blood loss may be the reason behind this result [18–20]. Our study suggested that patients with lower BMIs had a higher risk of transfusion. Patients with higher BMIs have a higher blood volume. Therefore, the percentage of perioperative blood loss in these patients is relatively low compared with that of patients with lower BMIs. In addition, we found that age was an independent risk factor for postoperative blood transfusion in patients with TKA, although it was not a significant factor in univariate analysis. Previous studies for elective arthroplasty have reported similar results [12, 15, 21–23].

Interestingly, with a strict statistical analysis, the results showed that coronary heart disease is one of the risk factors for postoperative blood transfusion in patients following TKA, and this finding has rarely been reported in previous studies. Recently, XU et al. identified that coronary heart disease is one of the risk factors for transfusion in patients following total joint arthroplasty [24]. In addition, Malcherczyk et al. [25] reported that coronary heart disease is a risk factor for postoperative blood transfusion in patients with shoulder arthroplasty. However, the potential mechanism between coronary heart disease and transfusion remains unclear. A possible explanation for this phenomenon is that patients with multiple diseases are more likely to use drugs that affect blood coagulation and have a higher risk of anemia [26]. In addition, the role of anti-inflammatory or antiplatelet drugs in blood management cannot be ignored. Recent meta-analyses showed that early hip fracture surgery was significantly related to a higher transfusion rate in patients with antiplatelet treatment than patients without antiplatelet treatment [27]. Therefore, antiplatelet drugs may play a role in influencing blood administration for TKA patients, and we will further study this potential mechanism in a later study. In addition, recent meta-analyses reported that patient-specific instrumentation decreased blood loss, but did not decrease transfusion rate [28]. Therefore, the complex relationship among coagulation, blood loss, and blood transfusion also need to be further studied.

Based on a large cohort, a preoperative nomogram was established and successfully validated in an independent cohort. All variables incorporated in the nomogram were easy to determine. By calculating the points of each of the five preoperative variables, arthroplasty surgeons can easily estimate the risk of postoperative transfusion before surgery. According to the evaluation results, the preoperative management strategy can be specified to reduce the risk of transfusion for high-risk patients. Similarly, for low-risk patients, some preventive measures could be reduced to decrease the economic burden and the risk of side effects [29].

There are also some limitations in our study. First, this is a retrospective study, so there may be inherent selection biases. However, we included as many preoperative factors as possible based on a large sample size to minimize biases. Second, although this nomogram has been verified by an independent cohort, we must realize that different blood transfusion policies in different hospitals, states and countries may limit the application of this nomogram to a few hospitals. Finally, although this nomogram showed excellent predictive power for postoperative blood transfusion, we must recognize the effect of surgical procedures on postoperative transfusion. Operation time, intraoperative blood loss, and intraoperative hemostasis are important factors influencing the transfusion rate in patients with TKA. Further prospective multicenter studies should be performed to further validate our nomogram.

## Conclusion

This study identified that lower preoperative Hb levels, simultaneous bilateral TKA, lower BMI, older age, and coronary heart disease are independent risk factors for postoperative transfusion in patients following TKA. A nomogram incorporating the above five predictors could accurately predict the transfusion risk.

## Supplementary information

**Additional file 1 Supplementary Fig. 1:** The results of LASSO regression analysis.

**Additional file 2 Supplementary Fig. 2:** The ROC curve showed the subgroup analysis of nomogram based on the categorical or the median of independent predictors. Training set(A-J); testing set(K-T). ROC: receiver operating characteristic; AUC: Area under the curve; CHD: Coronary heart disease; Hb: Hemoglobin; BMI: Body mass index.

**Additional file 3 Table 1**. Point of variables in the nomogram.

## Data Availability

The datasets generated during and/or analyzed during the current study are available from the corresponding author upon reasonable request.

## Abbreviations

TKA: Total Knee Arthroplasty
AUC: Area Under the Curve
DCA: Decision Curve Analysis
BMI: Body Mass Index
CCI: Charlson Comorbidity Index
TXA: Tranexamic Acid
RET: Reticulocyte
MCV: Mean Corpuscular Volume
MCH: Mean Corpuscular Hemoglobin
MCHC: Mean Corpuscular Hemoglobin Concentration
Hb: Hemoglobin
RDW: Red Blood Cell Distribution Width
PLT: Platelet
PDW: Platelet Distribution Width
