# Supplementary figures and images for: Development and validation of a nomogram to predict perioperative blood transfusion in patients undergoing total knee arthroplasty
Source: BMC Musculoskelet Disord. 2020 May 20;21:315. doi: 10.1186/s12891-020-03328-9 (PMC7241000; doi:10.1186/s12891-020-03328-9)

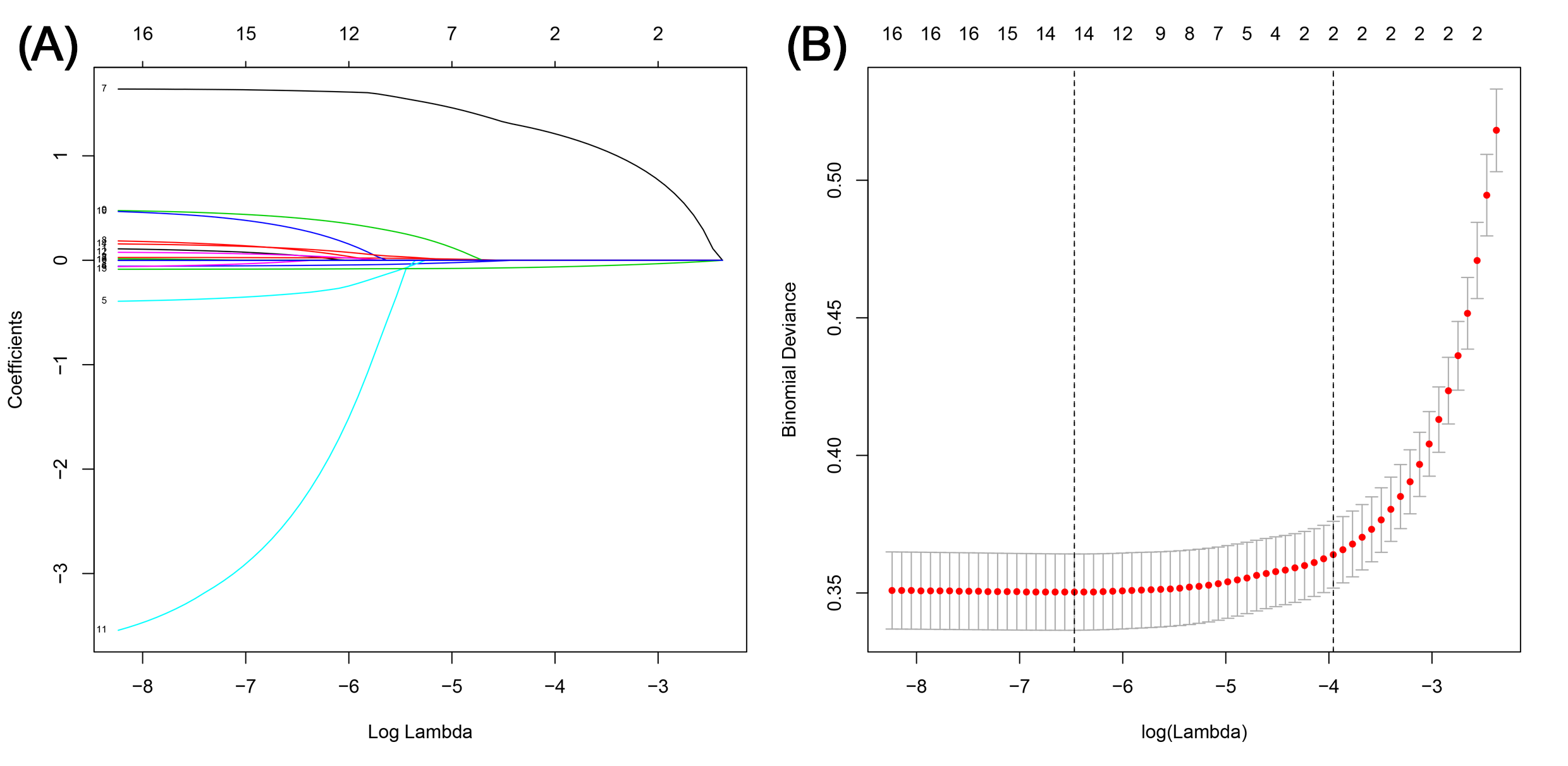

Supplement: Supplementary file 1 — Additional file 1 Supplementary Fig. 1: The results of LASSO regression analysis. [file 12891_2020_3328_MOESM1_ESM.tif]

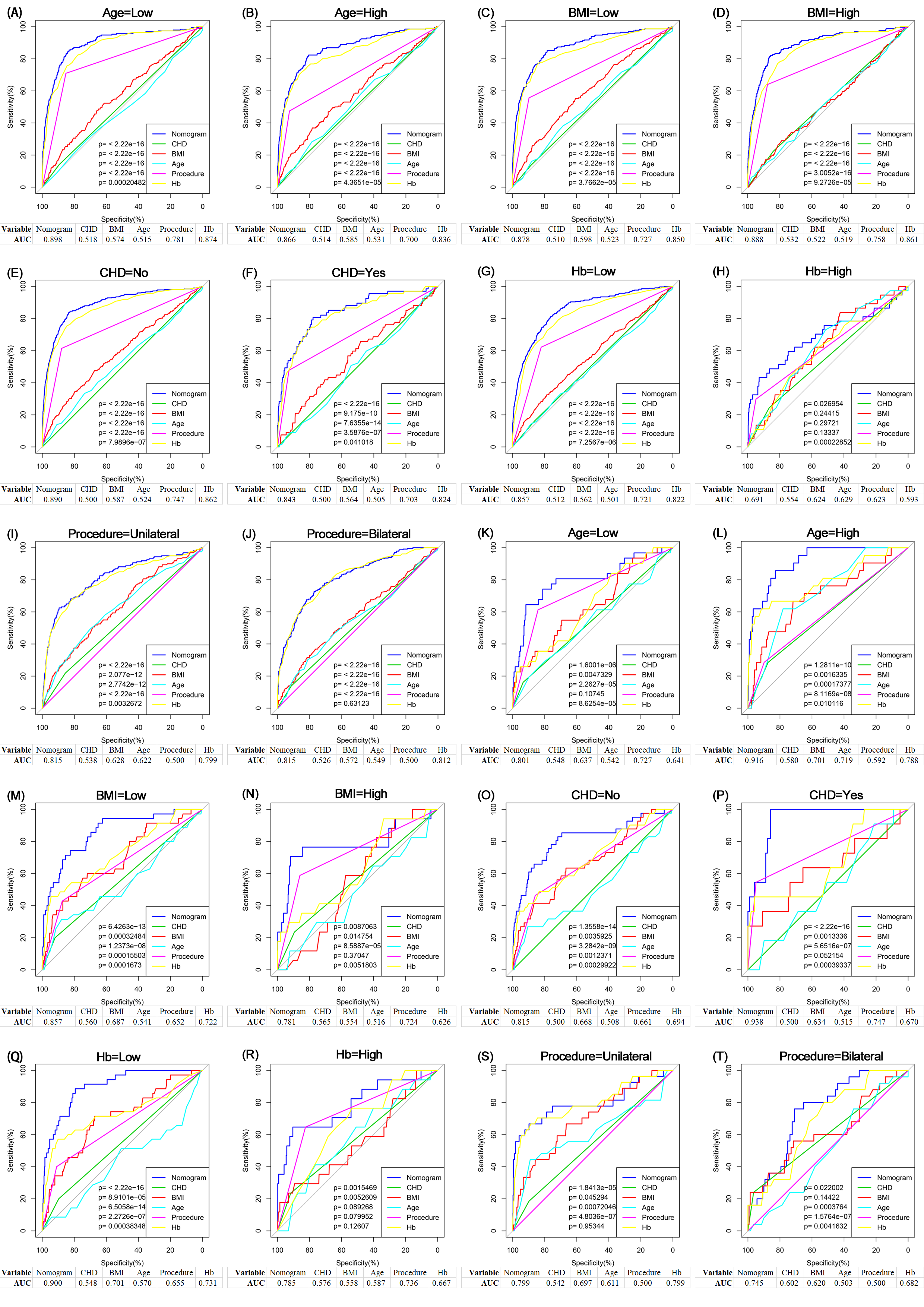

Supplement: Supplementary file 2 — Additional file 2 Supplementary Fig. 2: The ROC curve showed the subgroup analysis of nomogram based on the categorical or the median of independent predictors. Training set(A-J); testing set(K-T). ROC: receiver operating characteristic; AUC: Area under the curve; CHD: Coronary heart disease; Hb: Hemoglobin; BMI: Body mass index. [file 12891_2020_3328_MOESM2_ESM.tif]
