# Supplementary material for: Development and validation of a nomogram to predict perioperative blood transfusion in patients undergoing total knee arthroplasty
Source: BMC Musculoskelet Disord. 2020 May 20;21:315. doi: 10.1186/s12891-020-03328-9 (PMC7241000; doi:10.1186/s12891-020-03328-9)
Supplement: Supplementary file 3 — Additional file 3 Table 1. Point of variables in the nomogram. [file 12891_2020_3328_MOESM3_ESM.docx]

| Table 1. Point of variables in the nomogram | |
| --- | --- |
| Variable | Points or probability of transfusion |
| Age, year |  |
| 20 | 34 |
| 40 | 40 |
| 60 | 46 |
| 80 | 52 |
| 100 | 58 |
| CHD |  |
| No | 48 |
| Yes | 53 |
| BMI, kg/㎡ |  |
| 10 | 58 |
| 20 | 52 |
| 30 | 46 |
| 40 | 41 |
| Procedure |  |
| Unilateral TKA | 48 |
| Bilateral TKA | 65 |
| Hb, g/L |  |
| 80 | 68 |
| 90 | 59 |
| 100 | 51 |
| 110 | 42 |
| 120 | 34 |
| 130 | 25 |
| 140 | 16 |
| 150 | 8 |
| Total Points |  |
| 180 | 0.00% |
| 200 | 0.03% |
| 220 | 0.23% |
| 240 | 1.78% |
| 260 | 12.45% |
| 280 | 52.81% |
| 300 | 89.80% |
| 320 | 98.58% |

TKA: Total knee arthroplasty; CHD: Coronary heart disease; BMI: Body mass index; Hb: Hemoglobin
